# Health system and patient-level factors serving as facilitators and barriers to rheumatic heart disease care in Sudan

**DOI:** 10.1186/s41256-021-00222-2

**Published:** 2021-10-02

**Authors:** Jeffrey G. Edwards, Michele Barry, Dary Essam, Mohammed Elsayed, Mohamed Abdulkarim, Basamat M. A. Elhossein, Zahia H. A. Mohammed, Abdelmunim Elnogomi, Amna S. E. Elfaki, Ahmed Elsayed, Andrew Y. Chang

**Affiliations:** 1 Stanford University School of Medicine, Stanford, CA USA; 2 Present Address: Boston Medical Center, Department of Pediatrics, Boston University School of Medicine, Residency Program Coordinator, c/o Jeffrey Edwards, 801 Albany, St Boston, MA 02119-2598 USA; 3 Boston Children’s Hospital, Harvard Medical School, Boston, MA USA; 4 Department of Medicine, Stanford University, Stanford, CA USA; 5 Center for Innovation in Global Health, Stanford University, Stanford, CA USA; 6 Alazhari Health Research Center, Alzaeim Alazhari University, Khartoum, Sudan; 7 Faculty of Medicine, Alzaeim Alazhari University, Khartoum, Sudan; 8 Medical Technical College, Alzaeim Alazhari University, Khartoum, Sudan; 9 Department of Psychiatry, Alzaeim Alazhari University Khartoum, Khartoum, Sudan; 10 Al-Shaab Teaching Hospital, Khartoum, Sudan; 11 Cardiovascular Institute, Stanford University, Stanford, CA USA

**Keywords:** Rheumatic heart disease, Health services research, Global health, Risk factors, Barriers and facilitators to care

## Abstract

**Background:**

Rheumatic heart disease (RHD) remains a leading cause of morbidity and mortality in Sub-Saharan Africa despite widely available preventive therapies such as prophylactic benzathine penicillin G (BPG). In this study, we sought to characterize facilitators and barriers to optimal RHD treatment with BPG in Sudan.

**Methods:**

We conducted a mixed-methods study, collecting survey data from 397 patients who were enrolled in a national RHD registry between July and November 2017. The cross-sectional surveys included information on demographics, healthcare access, and patient perspectives on treatment barriers and facilitators. Factors associated with increased likelihood of RHD treatment adherence to prophylactic BPG were assessed by using adjusted logistic regression. These data were enhanced by focus group discussions with 20 participants, to further explore health system factors impacting RHD care.

**Results:**

Our quantitative analysis revealed that only 32% of the study cohort reported optimal prophylaxis adherence. Younger age, reduced primary RHD healthcare facility wait time, perception of adequate health facility staffing, increased treatment costs, and high patient knowledge about RHD were significantly associated with increased odds of treatment adherence. Qualitative data revealed significant barriers to RHD treatment arising from health services factors at the health system level, including lack of access due to inadequate healthcare staffing, lack of faith in local healthcare systems, poor ancillary services, and patient lack of understanding of disease. Facilitators of RHD treatment included strong interpersonal support.

**Conclusions:**

Multiple patient and system-level barriers to RHD prophylaxis adherence were identified in Khartoum, Sudan. These included patient self-efficacy and participant perception of healthcare facility quality. Strengthening local health system infrastructure, while enhancing RHD patient education, may help to improve treatment adherence in this vulnerable population.

**Supplementary Information:**

The online version contains supplementary material available at 10.1186/s41256-021-00222-2.

## Background

Rheumatic heart disease (RHD) is a chronic cardiovascular condition whose complications are preventable; yet it is responsible for an estimated 10.5 million disability-adjusted-life-years (DALYs) lost and over 300,000 deaths worldwide each year [1]. The majority of these mortalities occur in developing countries, with low and middle income countries (LMICs) accounting for most of the deaths from RHD [1–3]. It is the second leading cause of heart failure in children and young adults, as well as the third leading cause of heart failure for adults in Sub-Saharan Africa (SSA) [4–7]. The high prevalence of RHD in SSA, which accounts for half of the pediatric cases of RHD worldwide, despite having only 10% of the world’s population, remains in stark contrast to the low prevalence of the disease in high income countries [5].

Group A streptococcus (GAS) infection is the root cause of RHD, as it induces an abnormal immune response to the organism [2]. Without adequate treatment, GAS infections can cause acute rheumatic fever (ARF), a serious complication that causes inflammation and fibrosis of cardiac structures, including the valves, myocardium, pericardium, and conduction system. The cumulative injury from recurrent episodes of ARF is classified as RHD. To avoid recurrent episodes of ARF from repeated GAS infections, patients with RHD are advised to take prophylaxis in the form of intramuscular benzathine penicillin G (BPG) monthly for either ten years or until the patient turns 21 years of age (whichever is longer), with some guidelines suggesting lifelong prophylaxis [8–10].

Secondary prophylaxis has proven to be effective in preventing progression of disease in RHD patients [9, 11, 12]. Despite the reported efficacy of BPG, a multicenter RHD survey across 14 LMICs (the REMEDY registry study) reported that over 20% of enrolled patients did not regularly receive a monthly dose [3]. Previous studies in LMIC settings have suggested potential factors associated with low treatment adherence in RHD patients, including urban versus rural setting, education level, pain associated with injections, and availability of transportation funds [13]. Other qualitative analyses eliciting attitudes towards treatment and barriers to secondary prophylaxis found that key impediments to adherence included lack of resources (transportation, medications, clinic availability), injection pain, and poor communication between patients and providers [14].

In Sudan, there have been small decreases in annual deaths and DALYs lost due to RHD since 1990 [1, 15]. However, RHD still remains a major preventable cause of disability and mortality in the country [16, 17]. Low adherence rates to secondary BPG prophylaxis in populations with RHD continue to be reported and may be associated with the high rates of preventable heart valve injury in the country associated with the disease. Unfortunately, Sudan has low rates of cardiothoracic surgical availability (estimated to be as low as 7%) and secondary prophylaxis rates (estimated to be as low as 51%) for RHD, suggesting there is room for improvement in the nation’s RHD care intrastructure [16, 18]. This is compounded by the fact that there is inadequate knowledge of nationwide adherence rates, particularly among adults, due to a lack of adequate cases finding and monitoring in national public hospitals and registries. Furthermore, there is a paucity of literature describing the causes for these low observed adherence rates in the country.

Generally, RHD care takes place within the health system of Sudan, which is decentralized with a three-tier organization (federal, state, and district/locality) [19]. Most of the primary care health services are delivered via public hospitals or clinics, whereas secondary and tertiary care is more evenly divided between the private and public sectors [19]. Over one-third of Sudan’s health workforce can be found in Khartoum, the capital city [19]. There are 1.23 skilled health workers (medical doctors, nurses and midwives) per 1000 population, below the World Health Organization’s goal of 2.28 skilled health workers per 1000 population [19]. Out-of-pocket spending constitutes 70% of health care expenditures in Sudan (a high proportion compared to peer countries in North Africa) and disproportionately impacts Sudan’s low income population [19–21]. Further, there is no universal health coverage, with the National Health Insurance Scheme covering only 8% of the population [19].

Understanding healthcare phenomena such as treatment adherence, however, involves exploring not just health system factors, but those stakeholders and their relationships. Such an approach relies upon the health system dynamics framework [22], which posits that because there are necessary interactions between different levels of a health system, investigating these interfaces is important for developing meaningful medical interventions [22]. Factors that further influence these interactions include geographic, economic, and cultural access features [23]. By incorporating this multifaceted scheme, the health system dynamics framework has successfully been utilized to improve the quality of care from patient-, provider-, and healthcare facility-level perspectives in chronic diseases such as HIV (human immunodeficiency virus) and diabetes [24, 25]. We therefore believed that a more comprehensive understanding of health system and patient level factors associated with RHD treatment would help us to identify the facilitators and barriers to RHD care in Sudan. This knowledge could then effectively be used to target the root causes of low adherence and help providers limit further complications of RHD.

## Methods

### Study setting

The study was conducted in Khartoum, the capital of Sudan, a North- and Sub-Saharan African country that experienced an estimated 37,910 DALYs lost due to RHD in 2017 (the year the present study was conducted) [15]. Notably, accurate epidemiologic data surrounding the prevalence and incidence of RHD in Sudan remain uncertain, with a previous estimate for the incidence rate in Sudan being 100 per 100,000 population from the World Health Organization in 2001 [10].

The Khartoum metropolitan area is home to approximately 7.6 million of the country’s 40 million people [26]. Despite comprising less than 20% of the country’s population, the Khartoum metro houses a disproportionate degree of the country’s advanced health infrastructure, with 22 out of the 68 specialist hospitals located there [27]. This discrepancy is more pronounced in the field of cardiovascular medicine, where three of the four Sudanese hospitals providing cardiology care are found in Khartoum. The present study was conducted in two of these facilities: Al-Shaab Teaching Hospital, a 300-bed cardiology, chest medicine, and cardiothoracic surgery hospital; and Ahmed Gasim Cardiac and Renal Transplant Teaching Hospital, a 150 bed hospital providing cardiology, cardiac surgery and renal transplantation services.

### Study design

We chose to address our research question with a mixed-methods study, as we were interested in identifying actionable factors serving as barriers and facilitators to medication compliance. Thus, we felt that a convergent design with merged integration would allow us to query a priori assumed key factors by using a quantitative questionnaire, then simultaneously compare these findings with qualitative focus group responses [28]. Furthermore, we felt that focus groups would allow for inductive generation of additional compliance-affecting influences we had not anticipated in our quantitative study. The mixed-methods design would also allow for the quantification of the effect size of such factors by using survey response counts and better contextual understanding of the patient experience framing the importance of such factors.

### Quantitative

The quantitative portion of the study employed a cross-sectional design by utilizing patient surveys. These surveys included data on demographics (age, household income, education level), healthcare access (distance from facility, insurance status), and opinions on treatment barriers (Additional file 1). The primary objective variable was optimal benzathine penicillin adherence, defined as survey responses that indicated monthly BPG prophylaxis based on the prior 6 months. Patients and the public were involved in the study design and actively consulted during the study by providing the list of most common barriers to care for RHD patients, participating in a pilot survey, and ensuring that the research was culturally appropriate. We estimated that at a literature-based prevalence of 50% BPG nonadherence in the Sudanese RHD population, that a total cohort of 334 subjects would be necessary to detect a risk ratio of 1.3 (corresponding to an odds ratio of 1.8) for the objective variable between two groups (at significance level of 0.05 and power of 80%) [29]. We anticipated a 20% nonresponse rate, and aimed for a sample size of 400. A consecutive sampling strategy was deemed appropriate given the highly unique nature of the RHD patient experience compared to the general population [30]. Modified STROBE (Strengthening the Reporting of Observational Studies) guidelines were used to ensure proper reporting of methods and results (Additional file 2) [31].

### Qualitative

The qualitative portion of the study consisted of focus group discussions framed through a critical realist ontological perspective and a subjectivist epistemological stance (adopting a constructionist paradigm). These were chosen as we felt that there were physical and emotional factors that may strongly impact the outcomes of binary decisions in a behavioral fashion, but that the individual-level experience of such factors in the decision-making process is heavily influenced by complex societal considerations [32, 33].

To this end, four focus groups were administered at the study sites by two female Sudanese psychologists who were trained in qualitative research methods in groups of 3–4 patients and/or family members in Arabic by using a prepared focus group discussion guide (Additional file 3). A priori themes based on the literature were used to generate the first version of the document, which included specific programmatic and more general barriers and facilitators of receiving RHD treatment, specifically focusing on BPG therapy. No prior relationship existed between the focus group facilitators and participants, however, individual and study goals were shared with participants prior to the start of the study. The final sample size for the qualitative analysis was determined based on likelihood of achieving thematic saturation. However, based on general guidelines and reported ranges of grounded theory study sample sizes, we aimed to recruit 20–25 participants [34–40]. A convenience sampling strategy was deemed appropriate given the highly unique nature of the RHD patient experience compared to the general population [30]. COREQ (Consolidated criteria for Reporting Qualitative research) guidelines were used to ensure proper reporting of methods and results (Additional file 4).

### Study population

To characterize the demographics, socioeconomic status, BPG treatment adherence rates, and major RHD comorbidity burden of our study settings’ patient population, we first collected survey data from 397 patients aged 12 to 90 years who were enrolled in a regional RHD registry maintained by Alzaeim Alazhari University in Khartoum. The population included all patients within the university hospital catchment areas of Al-Shaab Hospital and Ahmed Gasim Hospital. Survey participants were selected via consecutive sampling among admitted patients or individuals attending routine clinic visits between July and November 2017 [41]. Participants were approached at the end of their hospital admission (if inpatient) or their clinic visit (if outpatient) to be recruited to the study.

### Data collection

Surveys were subsequently administered during that hospital admission or after clinic appointments by Sudanese medical students or physician trainees in English or Sudanese Arabic depending on participant preference.

To enrich our analysis with data triangulation by identifying key themes not captured by our surveys (which primarily represented a deductive approach), we conducted four focus group discussions with 20 patients and patient family members aged 20–66 years. Focus group participants were selected via consecutive sampling that occurred during clinic visits. All participants gave written consent to be included in the analysis. The study was conducted with Institutional Review Board (IRB) approval from the National Research Ethics Review Committee of Alzaeim Alazhari University (No. 4-5-2017), and Institutional Review Board of Stanford University (Protocol #40884).

### Data analysis/processing

#### Quantitative

Demographic variables and survey responses were described in counts, medians, and proportions (%). Since six injections in the preceding 6 months would indicate monthly BPG prophylaxis (standard treatment), participants were classified as not optimally adherent if they reported fewer than six BPG injections and adherent if they reported six or more injections. As a clinical variable, history of carditis was assessed by having medically trained research team members describe carditis to participants and then determining if clinical criteria were met. To assess which demographic and healthcare system factors were associated with an increased likelihood of adherence, we first constructed unadjusted binomial logistic regressions with each survey item as the independent variable. To control for major demographic confounding factors, we constructed an adjusted logistic regression model by using forward stepwise variable selection with a significance cutoff of *p*-value < 0.05. A stepwise selection algorithm was chosen due to the large number of candidate health system covariates considered for inclusion as model predictors and thus a desire to maintain investigator neutrality regarding their likelihood of significance. Robustness of the adjusted model was assessed using the Hosmer–Lemeshow goodness-of-fit test and area under the receiver operating characteristic curve (ROC). A *p*-value of < 0.05 was used to determine statistical significance regarding hypothesis testing. All quantitative statistical analyses were completed using Stata-SE, version 16.1 (College Station, TX).

#### Qualitative

Focus group discussion data were translated from Arabic into English by the research team and coded using the Dedoose qualitative analysis package (Los Angeles, CA) and Microsoft Word (Redmond, WA). Two independent readers from the analysis team (JE, AC) who were trained in qualitative research methods reviewed the transcripts and subsequently compared coding for the purposes of internal validity. Differences in coding were resolved through discussion. From this coding process, key themes and concepts were identified and classified by using the methodological orientation of grounded theory (GT), which was chosen as we wished to generate an integrated, comprehensive theory inductively from the data, in contrast to the deductively reasoned a priori*-*defined concepts utilized in the quantitative surveys [42]. Under the GT framework, the analysts identified potential organizing concepts in an initial reading and coding of the transcript, then iteratively reassigned codes and collapsed them into categories through abduction and constant comparative analysis in focused and theoretical coding phases. The process was continued until thematic saturation was achieved.

## Results

### Quantitative

#### Demographic factors

The demographic distribution of the surveys (Table 1) revealed that participants were mostly female (74.8%) and their ages ranged from 12 to 90 (median 40) years. Most of the respondents were homemakers or unemployed (72.5%) and had limited formal education, with the majority reporting their highest level of education as primary school or no formal schooling (66.7%). Nearly all participants (94.7%) reported a household monthly income less than 4000 Sudanese pounds (SDG; ~ 88 US dollars) with a median household size of six people. Over a quarter (28.7%) of participants self-reported that they had a history of carditis, though 51.4% of participants claimed to have had heart valve surgery. Only 32% of participants were found to be optimally adherent to BPG prophylaxis.

**Table 1:** Demographic and RHD clinical characteristics of quantitative survey respondents (N = 397)

*Gender*	n (%)
Female	297 (74.8%)
Male	100 (25.2%)
*Highest level of education*	
No formal schooling	124 (31.2%)
Primary school	141 (35.5%)
Secondary school	84 (21.2%)
University	48 (12.1%)
*Employment status*	
Homemaker	220 (55.4%)
Employed	91 (22.9%)
Unemployed	68 (17.1%)
Student	18 (4.5%)
*Monthly income level*	
Less than 2000 Sudanese Pounds (SDG) *(100 SDG = $15.06 USD)	275 (69.3%)
2000–3999 SDG	98 (24.7%)
4000–7999 SDG	19 (4.8%)
Greater than 8000 SDG	2 (0.5%)
Not reported	3 (0.7%)
*Household setting*	
Rural	179 (45.2%)
Urban	175 (44.2%)
Suburban	42 (10.6%)
*Insurance status*	
Insured	278 (70.0%)
Uninsured	107 (27.0%)
Not reported	12 (3.0%)
*History of carditis*	
Yes	114 (28.7%)
No	283 (71.3%)
*History of heart valve surgery*	
Yes	204 (51.4%)
No	193 (48.6%)
*Optimal adherence to BPG prophylaxis*	
Yes	127 (32.0%)
No	270 (68.0%)

Demographic and clinical characteristics of the 397 patients with RHD who completed a quantitative survey outlining barriers to BPG prophylaxis in Khartoum, Sudan

#### Association of factors with penicillin prophylaxis adherence

In unadjusted analyses, younger subject age, female gender, higher household monthly income, higher educational level, rural residence (relative to suburban), shorter healthcare facility wait times, perceived adequate staffing at healthcare facility, treatment costs, and patient understanding of disease were all identified as factors significantly associated with optimal prophylactic BPG adherence (Table 2).

**Table 2:** Factors associated with BPG prophylaxis adherence in quantitative survey participants

Variable	Unadjusted analysis		Adjusted analysis	
	Odds ratio	*P* value	Odds ratio	*P* value
Age (per year)^a^	0.98 (0.97–1.00)	0.020*	0.973 (0.952–0.995)^b^	0.015
Female gender (Ref male)	1.94 (1.14–3.29)	0.014*	^	^
*Employment status*				
Employed	Reference		Reference	
Unemployed	0.90 (0.45–1.82)	0.770		
Homemaker	1.37 (0.81–2.34)	0.243	^	^
Student	0.96 (0.31–2.97)	0.946		
*Household monthly income*				
< 2000 SDG (Sudanese Pound)	Reference		Reference	
2000–3999 SDG	2.02 (1.26–3.25)	0.004*	^	^
4000–7999 SDG	0.66 (0.21–2.06)	0.475	^	^
> 8000 SDG	-	-	-	-
*Educational level*				
No formal schooling	Reference		Reference	
Primary School	1.25 (0.73–2.13)	0.412	^	^
Secondary School	1.45 (0.80–2.65)	0.222	^	^
University	2.15 (1.07–4.30)	0.032*	^	^
# of People in Household^a^	1.00 (0.94–1.07)	0.931	^	^
# of Rooms in Household^a^	1.14 (0.99–1.32)	0.069	^	^
*Urban/rural residence*				
Rural	Reference		Reference	
Urban	1.20 (0.78–1.87)	0.409	^	^
Suburban	0.36 (0.14–0.90)	0.028*	^	^
Insured status (Ref uninsured)	0.90 (0.56–1.44)	0.658	^	^
History of carditis (self-reported)^c^	**1.36** (0.86–2.15)	0.189	^	^
History of heart valve surgery	1.25 (0.82–1.90)	0.308	^	^
Distance to healthcare facility in km^a^	0.997 (0.994–1.000)	0.051	^	^
Wait-time at healthcare facility (per minute)^a^	0.997 (0.994–0.999)	0.004*	0.995 (0.992–0.999)^b^	0.006
Perceived adequate staffing at healthcare facility (ref inadequate staffing)	2.07 (1.16–3.71)	0.014*	3.47 (1.48–8.17)^b^	0.004
**Transportation costs**^**a**^	0.999 (0.997–1.001)	0.469	^	^
Treatment costs (per Sudanese Pound)^a^	1.013 (1.002–1.025)	0.022*	1.015 (1.002–1.028)^b^	0.025
Reported primary barrier to treatment				
None listed	Reference		Reference	
Cost of medicine/treatment	0.60 (0.23–1.56)	0.293	^	^
Cost of travel to receive care	0.47 (0.15–1.45)	0.188	^	^
Distance to nearest healthcare facility	0.61 (0.20–1.86)	0.387	^	^
Fear of injection pain	0.38 (0.10–1.40)	0.145	^	^
Lack of understanding of RHD	0.22 (0.08–0.56)	0.002*	0.32 (0.16–0.62)^b^	0.001

a Continuous variable

b Adjusted for age, treatment costs, and wait-time at healthcare facility

c History of carditis was self-reported with clinical expertise assistance from research team members

^*^p value < 0.05 in unadjusted analysis

^ Excluded from final model by variable selection procedure

- Insufficient data for regression

Unadjusted and adjusted odds ratios for factors associated with BPG prophylaxis in a cohort of 397 patients with RHD who completed a quantitative survey in Khartoum, Sudan

Following final model variable selection and adjustment, however, age, healthcare facility wait time, perceived adequacy of healthcare facility staffing, treatment costs, and lack of understanding of RHD remained significantly associated with likelihood of optimal BPG adherence. For every year increase in subject age, there was a 2.7% decrease in odds of adherence (Odds Ratio (OR) = 0.973; 95% CI 0.952–0.995). Similarly, for every minute increase in wait time at the subject’s RHD care facility, there was a 0.3% decrease in odds of adherence (OR = 0.997, 95% CI 0.994–0.999). Study participants who felt that there was adequate staffing at their healthcare facility had over a two-fold increase in likelihood of BPG adherence (OR = 3.472, 95% CI 1.475–8.172). Meanwhile, patients who reported a lack of understanding of RHD as their primary barrier to appropriate treatment were 78% less likely to be adherent to BPG (OR = 0.319, 95% CI 0.164–0.619). Interestingly, for every 1 SDG (~ 0.15 USD in 2017) increase in treatment cost, there was a 1.5% increase in odds of adherence (OR = 1.015; 95% CI 1.002–1.028).

#### Qualitative analysis

**Fig. 1:**
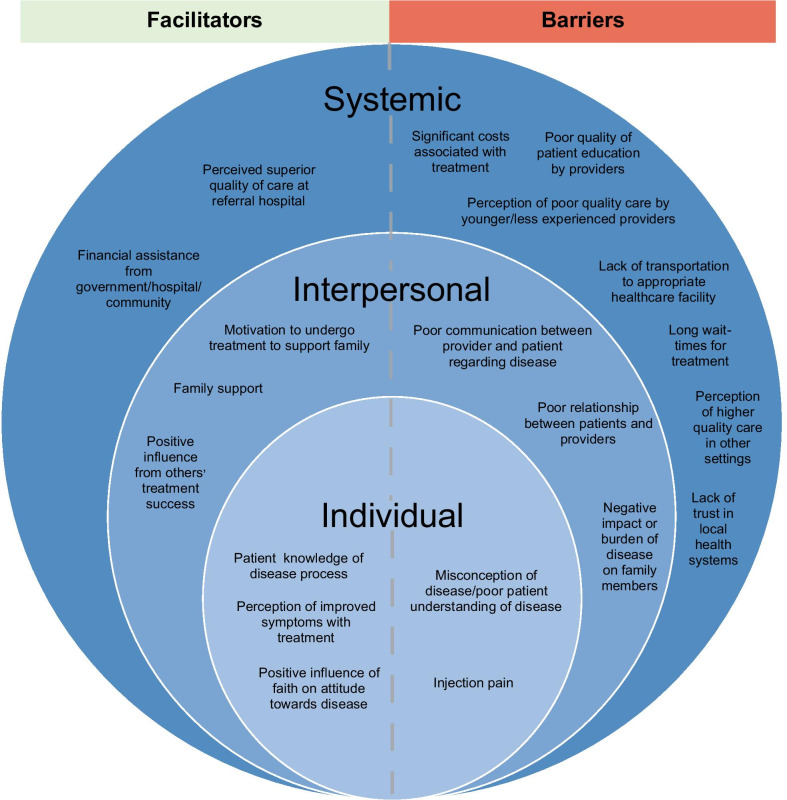
Major themes of focus group discussions. The major themes that emerged from the focus group discussions with patients and their family members, grouped together by domain (individual, interpersonal, or health system-level) and classified as a facilitator or barrier

Twenty individuals aged 20–66 (median age of 41) years were represented in four focus groups, with eleven patients and nine patient family members. 55% were female; 55% reported a monthly income level less than 2000 SDG; and 75% of participants lived in a rural setting (Additional file 5, Table 1). Of the eleven patients, four (36.4%) had undergone valve surgery. Analysis of the focus group discussions revealed recurring major and minor themes that were categorized as facilitators or barriers to receiving treatment in one of three domains: individual, interpersonal, or health system factors (Fig. 1).

### Facilitators of appropriate therapy

Multiple facilitators of obtaining appropriate RHD treatments were identified from our focus group discussions, with elements attributable to individual, interpersonal, and health system factors (Table 3). Individual facilitators included adequate knowledge of RHD by the patient, perception of improved symptoms with treatment, and the positive influence of faith on attitude towards treatment. For example, one patient described an intimate understanding of the dietary factors influencing the therapeutic and supratherapeutic levels of their blood-thinning medications, allowing them to take control of their own healthcare in this regard. Another reported compliance with prophylactic BPG to avoid prior symptoms. A third reported their faith as a reason to approach surgical management with optimism (Additional file 6, Table 2A). Interpersonal facilitators of treatment, meanwhile, included a motivation to undergo treatment as a means of supporting family members, multifactorial family support with treatment, and a positive influence from other’s treatment success. One male participant (Subject No. 7) recalled consistent support from their mother, stating “All of this was a result of my mother’s prayers. She fought hard for me, she is my whole family to me, she spent almost 20 years at the side of my hospital bed.” Another participant related their desire to pursue therapy to relieve their family of their care duties. Additionally, a subject reported being inspired by the ability of a neighbor to proceed with daily life following their RHD operation.

**Table 3:** Exemplar quotes from qualitative focus group discussions

Domain	Major theme	Exemplar quote
Individual	Patient knowledge of disease process	“If my anticoagulation levels were high I would eat some watercress [leafy vegetable] for 3 days and lower them to 2–2.5 if find they are 1–1.5 I would eat some ginger for 3 days and It will return to a 3…Once they were shocked and told me my INR is 10, I said ok, just leave me for a few days in which I ate lentils and liver. After 3 days I returned and they checked it…it was 2.5”
Interpersonal	Positive influence from other’s treatment success	“I see how [another patient] is living since she lives in our neighborhood… She is the one who is encouraging me to do the operation and get relieved from this stress…We had the wedding of her cousin in our same house and everybody was coming to say farewell to her. She was normal and the wedding proceeded normally and she did not suffer, God protected her”
Systemic	Perceived superior quality of care at referral hospital	“But honestly I see Ahmed Gasim hospital [a referral hospital] as one of the best hospitals and this a truth. The doctors are great and collaborative with the patients”
Individual	Misconception of disease/poor understanding of disease	“I mean drinking too much coffee and cigarettes. All these things cause complications and affect the heart because the heart is the main building which pushes the body. Also, too much stress affects the heartbeat, which affects the heart arteries”
	Injection pain	“Yes, it was painful and that was also a reason of stopping it, but I didn’t realize [stopping it] would deteriorate me to this stage”
Interpersonal	Poor communication between provider and patient	“Getting a definite date for surgeries is important. Especially for people who live in far areas. Meeting a doctor several times and not getting a definite date is frustrating. It would be very much comforting to be assigned a date at an early time”
Systemic	Lack of transportation to appropriate healthcare facility	“Transportation is a major problem between the hospitals and from the house to the hospital. Autumn is 3–4 months and because of the rain you can’t leave your house and get medical care, there is also the issue of the bus fee”

Exemplar quotes from the focus group discussions with 20 patients/patient family members with RHD. These quotes reflect a selection of the major and minor themes that emerged using grounded theory orientation. The full list of themes with additional exemplar quotes can be found in Additional file 2A and B

Health system facilitators of treatment adherence that emerged included a perceived superior quality of care at referral hospitals and financial assistance from the government or hospital facilities. Participants generally had positive experiences at referral hospitals, with one female participant (Subject No. 2) claiming, “…the healthcare here [Ahmed Gasim] is great, the doctors and the nurses are helpful and the bathrooms are always clean the whole 24 hours and there is no problem.” Interestingly, a number of our study participants also spontaneously noted that ancillary services such as friendliness of security personnel or cleanliness of lavatories as positive reinforcement of trust in their health center. For example, focus group members also praised Al-Shaab Hospital for its clean toilets and reliable access to meals (Additional file 6, Table 2A).

### Barriers to appropriate therapy

Individual barriers to receiving RHD treatment included misconception of the disease process or treatment, as well as pain from BPG injection. The various misconceptions regarding RHD included one female participant (Subject No. 9) stating that the cause of RHD was “…drinking too much coffee and cigarettes. All these things cause complications and affect the heart because the heart is the main building which pushes the body. Also, too much stress affects the heartbeat, which affects the heart arteries” (Additional file 6, Table 2B). One participant recalled stopping their penicillin injections due to the pain, not knowing that cessation of the prophylaxis would lead to RHD disease progression. Interpersonal barriers we identified included poor communication and poor relationship between patients and healthcare providers and the negative impact of the disease upon the patient’s family members. One focus group participant mentioned how frustrating it was to not be given a definitive date for their cardiac operation from their physician, a factor magnified by distance from the health center (Table 3).

Health system barriers to treatment adherence reported by our cohort were numerous, including poor quality of disease/treatment education by providers, significant costs associated with treatment across several domains, perception of lower quality care by younger/less experienced physicians, a lack of trust in local healthcare systems, a lack of transportation to appropriate healthcare facilities, long wait time associated with treatment, and a perception of higher quality care provision in other settings. Highlighting health system issues with transportation, one male participant (Subject No. 7) noted, “Transportation is a major problem between the hospitals and from the house to the hospital. Autumn is 3–4 months [long] and because of the rain you can’t leave your house and get medical care; there is also the issue of the bus fee.” Another pointed out the significant costs associated with housing near a referral hospital. Emphasizing the lack of trust some RHD patients had with local (non-referral) health centers, focus group respondents mentioned the lack of laboratories, specialists, experienced physicians, and continuity of care; hospital shutdowns for repairs; and misdiagnoses between local specialists as reasons for poor care (Additional file 6, Table 2B).

## Discussion

Our study offers a detailed survey of the barriers and facilitators to receiving adequate RHD care in the urban setting of Khartoum, Sudan. Although prior analyses have offered estimates of the epidemiologic characteristics of RHD, few have focused on the specific LMIC healthcare system barriers to treatment contributing to the disparate prevalence compared to that of high-income countries. This study’s strength lies in its mixed-methods design that bolsters the findings of our quantitative surveys with themes independently identified in our focus groups. Applying these methods to the relatively poorly studied region of Sudan allows us to identify targeted interventions for context-specific issues.

Our quantitative survey revealed a predominantly female population (75%) with low educational attainment, employment level, and monthly income. These values are consistent with other RHD epidemiologic surveys elsewhere in SSA [43]. Interestingly, our sample reported low rates of optimal adherence to prophylactic BPG, with only 32% calculated from self-report, to meet adherence metrics in the previous six months. These values are lower than those found in prior studies of Sudanese populations, but these analyses represented pediatric cohorts, which often experience higher linkage and retention in clinical care [18, 44].

### Patient-level barriers to RHD care

As for patient-level barriers identified in the survey, a lack of understanding of RHD was identified as the primary barrier to receiving adequate care that was most strongly associated with poor BPG adherence. This finding was reinforced by the major themes of poor disease education quality and poor communication between patients and providers in the focus groups. Though this appears to be a robust factor, poor education on RHD can be addressed in a multitude of cost-effective ways. Patient education campaigns, public service announcements (PSAs), and healthcare provider training are all low-cost interventions that can improve patient understanding of disease and serve as both primary and secondary prevention of RHD. In particular, PSAs have been successful at increasing patient knowledge in LMICs in the Caribbean, though sociopolitical differences must be accounted for when adapting that strategy to SSA [12, 45]. Female gender and a higher household income were also found to confer a higher likelihood of adherence with RHD treatment in unadjusted analysis whereas suburban household setting was associated with a decreased likelihood of adherence, which provides insight into possible appropriate targeting of educational interventions. In Zambia, a public–private partnership exemplified the efficacy of targeted educational interventions based on the results of mixed-methods research, which serves as a model for designing interventions in Sudan from this project’s findings [46].

### Health system-level barriers to RHD care

Several health system barriers to treatment adherence identified in our study (high treatment costs and limited access to appropriate healthcare) overlapped with prior studies in Uganda [14]. Our analysis is unique, however, in eliciting patient attitudes toward health system factors, and we identified that perceived inadequate healthcare staffing was a robust barrier to optimal BPG adherence. In qualitative analyses, this was bolstered by our finding that ancillary health center services such as facility cleanliness, nutritional services, and security also impacted patient trust in the RHD care system. These factors, which appear to reflect patient perception of health facility quality, further outlines the long-term healthcare infrastructure shortfalls existing in Khartoum as related to care for chronic illnesses. Partnership with the Sudanese government can most feasibly be achieved through following the needs assessment tool for developing effective RHD programs, as outlined by Zühlke and colleagues [47]. Based on that approach, this study’s results combined with additional stakeholder interviews in Sudan could be used to design community-based interventions to increase patient trust in RHD healthcare services. Another approach to achieving policy change could involve guidance from cost-effectiveness models, which may inform policymakers of the prudent investment in primary and secondary prevention costs as compared to the workforce and surgical repair costs associated with severe RHD, as was proposed by researchers in Kenya [48].

Though transportation costs were not found to be a statistically significant factor associated with lower treatment adherence in our survey, transportation issues were highlighted as major themes throughout our focus group discussions. Given the large distances between many Sudanese towns and its capital, Khartoum, it is understandable that transportation remains a major barrier to receiving care, as identified by 17% of the cohort. Transportation issues could be alleviated through the use of mobile health clinics, such as those utilized to mobilize maternal health care in Sudan in 2015 [49]. Another approach would include the decentralization of RHD care at specialized district level centers, as was done in Uganda [43, 50, 51].

Similarly, though injection pain was only cited by 5% of participants as the primary barrier to treatment adherence, it emerged as a major theme in focus group discussions. Because of the higher efficacy of BPG injections over oral penicillin equivalents for secondary prevention, however, this is unlikely to be addressed outside of providing analgesia to those patients [52, 53]. Regarding primary prevention, further development of a previously studied GAS vaccine could provide primary prevention of GAS infections and its complications. Unfortunately, promising vaccine candidates have displayed cross-reactivity with human tissue and the high number of GAS subtypes makes vaccine development challenging at present [54–56].

### Facilitators of proper RHD care

Family support was revealed as a facilitator of treatment adherence in focus groups, with many participants noting that family members helped with treatment costs, transportation and lodging associated with referral hospital visits, while providing emotional support. This contrasts somewhat with the survey data, which suggested that 89% of respondents self-reported strong family support, even though the adherence rate in that cohort was 32%.

The positive experiences of RHD patients at referral hospitals can also serve as a template for local medical facilities providing care for RHD patients. Though many of the inadequacies of local facilities were not limited to RHD care, these centers can use this information to improve the perception of care quality by those in their communities, for example through the establishment of RHD Centers of Excellence, as has been done elsewhere in sub-Saharan Africa [57]. Moving forward, a comprehensive approach to RHD control, such as the SUR I CAAN program adopted in Sudan, can offer a practical model for addressing this multifactorial issue resource-limited settings [58].

## Limitations

There are several limitations to our current study. First, the use of an academic center-based registry population likely selected for an analysis cohort with a high symptom burden and advanced RHD disease state. Further, the study represented a consecutive sample of patients during the enrollment time period, presenting an opportunity for selection bias. This is partially mitigated by the large number of participants in the analysis and relative representativeness of demographic characteristics in our cohort that we encountered. Lastly, although this study’s findings would ideally be extrapolated to develop interventions for other resource-limited settings, the unique geopolitical situation of Sudan may limit the generalizability of this study.

## Conclusions

Our study demonstrates that significant facilitators and barriers to RHD care exist at the patient and health system levels in Sudan. Some notable facilitators include reduced healthcare facility wait times, strong interpersonal support at the patient level, and the perception of adequate staffing at the health system level. Significant barriers to RHD care include lack of understanding of RHD, and treatment/ transportation costs at the patient level, and poor quality of the healthcare infrastructure at the health system level. Our study is unique in highlighting the health system factors that are contributing to the suboptimal BPG adherence rates reported in Sudan. Interventions that target these identified barriers and strengthen facilitators could continue the trend of improvement in RHD outcomes in Sudan and work towards an improved cardiovascular health system in Sudan more broadly.

## Supplementary Information

**Additional file 1**. The survey intake form for the quantitative portion of this study. This survey collect demographic, clinical and disease-specific information from participants.

**Additional file 2**. The STROBE checklist for this study. This form identifies standardized information that should be present in all cross-sectional studies.

**Additional file 3**. The focus group interview guide for the qualitative portion of this study. This interview guide included initial and probing questions used to guide the focus group discussion.

**Additional file 4**. The COREQ checklist for this study. This form identifies standardized information that should be present in all qualitative studies.

**Additional file 5**. This is a supplemental table providing demographic information for the participants in the qualitative portion of this study.

**Additional file 6**. This is a supplemental table providing exemplar quotes from the qualitative portion of the study, alongside their corresponding major and minor themes. This provides a more in-depth look at the participant responses from the focus group discussions.

## Data Availability

The datasets used and/or analyzed during the current study are available from the corresponding author on reasonable request.

## Abbreviations

RHD: Rheumatic heart disease
LMICs: Low and middle income countries
SSA: Sub-Saharan Africa
GAS: Group A streptococcus
ARF: Acute rheumatic fever
BPG: Benzathine penicillin G
