# Supplementary material for: Health system and patient-level factors serving as facilitators and barriers to rheumatic heart disease care in Sudan
Source: Glob Health Res Policy. 2021 Oct 2;6:35. doi: 10.1186/s41256-021-00222-2 (PMC8486630; doi:10.1186/s41256-021-00222-2)
Supplement: Supplementary file 1 — Additional file 1. The survey intake form for the quantitative portion of this study. This survey collect demographic, clinical and disease-specific information from participants. [file 41256_2021_222_MOESM1_ESM.docx]

**Questionnaire on Rheumatic Heart Disease Treatment**

**Part 1: Demographics**

1. What is your age? [ ]
2. What is your gender?

☐ Male

☐ Female

1. What is your employment status?

☐ 1 Employed

☐ 2 Unemployed – Looking for Job

☐ 3 Homemaker (Housewife/Househusband)

☐ 4 Student

1. What is your household’s monthly income?

☐ 1 Less than 2,000 Sudanese pounds

☐ 2 2,000 to 3,999 Sudanese pounds

☐ 3 4,000 to 7,999 Sudanese pounds

☐ 4 8,000 Sudanese pounds or more

1. What is your educational level?

☐ 1 None (No formal schooling)

☐ 2 Primary School

☐ 3 Secondary School

☐ 4 Vocational (Trade) School

☐ 5 University

1. How many people live in your household? [ ]
2. How many rooms are in your household? [ ]
3. Would type of setting is your household?

☐ 1 Rural

☐ 2 Urban

☐ 3 Suburban

1. Do you have health insurance?

☐ 0 Yes ☐ 1 No

1. Have you had episodes of carditis (heart inflammation) in the past?

☐ 0 Yes ☐ 1 No

1. Have you had heart valve repair surgery related to rheumatic heart disease (RHD)?

☐ 0 Yes ☐ 1 No

1. How frequently do you receive benzathine penicillin G (BPG) treatment for RHD?

**Part 2: Access to Healthcare**

1. Do you have support from family members when seeking out healthcare?

☐ 0 Yes ☐ 1 No

1. How far are you from the nearest healthcare facilities (estimate distance in kilometers)? [ ]
2. How long is your average wait time at healthcare facilities (estimate time in minutes)? [ ]
3. Do you believe that your healthcare facilities have adequate staffing?

☐ 0 Yes ☐ 1 No

1. On average, how much do you spend on transportation costs (estimate in Sudanese pounds (SDG)) to reach healthcare facilities? [ ]
2. On average, how much do you spend on BPG costs (estimate in SDG)?* [ ]

**Part 3: Attitude Towards Treatment**

1. Do you believe there are significant barriers to receiving BPG treatment for RHD?

☐ 0 Yes ☐ 1 No

1. If so, which of the following are some of those barriers? (Check all that apply)

☐ 1 Cost of Medicine/Treatments

☐ 2 Cost of Travel

☐ 3 Distance from Health Centers/Pharmacies

☐ 4 Fear of Medications

☐ 5 Lack of Awareness of Severity of Disease

1. Do you believe that BPG treatment via monthly intramuscular injections is safe?*

☐ Yes ☐ No

1. Do have pain when receiving the monthly BPG injections?*

☐ Yes ☐ No

1. Are there any negative side effects you have after receiving the BPG injections?*

☐ Yes ☐ No

1. Are you aware of the complications that can result from untreated RHD?

☐ Yes ☐ No

If so, please list some below:

____________________________________________________________

1. Are you aware of the length of BPG treatment that is required for RHD?

☐ Yes ☐ No

If so, please specify the length below:

____________________________________________________________

1. Which of the following would you say is the primary barrier to care? (Choose one)

☐ 1 Cost of Medicine/Treatments

☐ 2 Cost of Travel

☐ 3 Distance from Health Centers/Pharmacies

☐ 4 Fear of Medications

☐ 5 Lack of Awareness of Severity of Disease

1. Is there anything else you would like to share with the researchers?
