# Supplementary material for: Health system and patient-level factors serving as facilitators and barriers to rheumatic heart disease care in Sudan
Source: Glob Health Res Policy. 2021 Oct 2;6:35. doi: 10.1186/s41256-021-00222-2 (PMC8486630; doi:10.1186/s41256-021-00222-2)
Supplement: Supplementary file 3 — Additional file 3. The focus group interview guide for the qualitative portion of this study. This interview guide included initial and probing questions used to guide the focus group discussion. [file 41256_2021_222_MOESM3_ESM.docx]

**Focus Group Interview Guide**

**Characteristics and Motivations of BPG Adherence in Patients with Rheumatic Heart Disease in Sudan: A Mixed Methods Study**

**Introductory remarks: To be given by the moderator**

Thank you for joining us today for this focus group. We are here today to learn from you how people living with heart disease feel about certain medications used to treat rheumatic heart disease. In particular, we are interested in your perspective as a patient with rheumatic heart disease in an urban setting in Sudan, since the Sudan Ministry of Health with cardiac conditions like rheumatic heart disease. We have been compiling data for a national registry, and wish to supplement this by having discussions with patients and community members to better serve your health needs.

You have been invited to participate in our study as a member of a “focus group”, a collection of patients of similar background. The format is not a group interview, but rather an opportunity for you to share your opinions and experiences on the topics we discuss today, ultimately allowing us to build a better health program.

There are several important rules for this meeting:

1. There are no right or wrong answers. We simply wish to hear your opinions on the matters we are to discuss.
2. All of the opinions and statements from today’s discussion must remain confidential and should not be repeated outside of today’s focus group.
3. Please do not discuss this meeting with people who were not present here at the meeting.
4. For your protection and to preserve anonymity, please choose a fake name which cannot be traced back to you. Every time you speak into the digital audio recorder, please state that “it is [your chosen fake name]” so that we know who is speaking when we review the record later.
5. The study team is the only group of people who will have access to the tape. Nobody outside this room will be able to trace your comments back to you.
6. Please speak one-by-one. Do not speak when someone else is talking. Also, speak clearly and loudly so the recorder can pick up what is said.

As the moderator, I am here to ask questions and listen, but I will not be participating in the discussion. Please feel free to discuss each question with one another. My role is to guide the discussion to collect the information most useful for the researchers.

My role, however, may mean that I will do certain things, including:

1. I may ask some participants who are quieter to volunteer their thoughts, and others who have many great ideas to space out your comments so everybody gets a chance to share their opinions.
2. Some topics of discussion may evoke strong emotions from participants. We ask that you respect the feelings of all members of the group here. Again, please protect the confidentiality of all speakers today regarding these subjects.
3. If the discussion begins to stray off the subject, I may ask that we re-direct and re-focus our conversation back to the study questions at hand.

**Discussion Questions (For Patients):**

**General Questions:**

1. Tell me what you know about your cardiovascular illness (rheumatic heart disease)—

Prompts: What caused it, how it is treated, and how does it impact your everyday life?

**Patient Perspective Questions:**

1. Has a doctor ever discussed asked you for your perspective on Rheumatic Heart Disease of using Benzathine Penicillin G as treatment?
   1. How would you describe your condition to others?
   2. How does having Rheumatic Heart Disease affect your daily life, if at all?
   3. What has your doctor told you about complications from Rheumatic Heart Disease?
   4. Do you feel there is a prejudice or judgment directed against people who have heart disease and continuously receive treatment for it?

**Attitudes Towards Healthcare Questions:**

1. Can you describe your general attitudes towards receiving healthcare?
   1. How has your diagnosis and treatment for Rheumatic Heart Disease changed your opinion of the quality of healthcare available to you?
   2. Do you believe that the healthcare facilities available to you are sufficient for treating your condition? If not, what steps would you recommend for these facilities?
   3. In what ways do you believe access to healthcare for patients with Rheumatic Heart Disease can be improved in your area?

**Attitudes Towards Treatment Questions:**

1. Can you describe your general attitudes towards receiving the Benzathine Peniciilin G treatment for Rheumatic Heart Disease?
   1. Can you talk about any fears you may have concerning the safety of intramuscular Benzathine Penicillin G injections for treatment?
   2. Can you describe any pain associated with receiving the Benzathine Penicillin G injection?
   3. Can you explain any adverse side effects or reactions associated with Benzathine Penicillin G treatment?
   4. Can you talk about any difficulty you may have maintaining monthly prophylaxis due to your schedule?

**Treatment Barrier Questions:**

1. Are there significant barriers to obtaining adequate treatment for people with rheumatic heart disease?

If so, what do you believe are the primary barriers?

- Cost of medicine/ treatments?

- Cost of travel?

- Distance from health centers/ pharmacies?

- Fear of medications?

- Judgment by family/ friends?

**Concluding Question:**

1. Can you share any other thoughts or concerns you have with the study coordinators?

Did we miss anything? Any other experiences you would like to share?
