# Supplementary material for: Health system and patient-level factors serving as facilitators and barriers to rheumatic heart disease care in Sudan
Source: Glob Health Res Policy. 2021 Oct 2;6:35. doi: 10.1186/s41256-021-00222-2 (PMC8486630; doi:10.1186/s41256-021-00222-2)
Supplement: Supplementary file 5 — Additional file 5. This is a supplemental table providing demographic information for the participants in the qualitative portion of this study. [file 41256_2021_222_MOESM5_ESM.docx]

**Supplementary Material 5: Supplementary Table 1**

**Table 1: Qualitative Focus Group Demographics (N=20)**

| **Gender** | | n (%) |
| --- | --- | --- |
|  | Female | 11 (55.0%) |
|  | Male | 9 (45.0%) |
| **Highest Level of Education** | | |
|  | No formal schooling | 8 (40.0%) |
|  | Primary school | 7 (35.0%) |
|  | Secondary school | 4 (20.0%) |
|  | University | 1 (5.0%) |
| **Employment Status** | | |
|  | Homemaker | 9 (45.0%) |
|  | Unemployed | 5 (25.0%) |
|  | Employed | 4 (20.0%) |
|  | Student | 2 (10.0%) |
| **Monthly Household Income** | | |
|  | Less than 2000 SDG | 11 (55.0%) |
|  | Greater than 2000 SDG | 9 (45.0%) |
| **Household Setting** | |  |
|  | Rural | 15 (75.0%) |
|  | Urban | 5 (25.0%) |
| **Focus Group Location** | |  |
|  | Al-Shaab Hospital | 10 (50.0%) |
|  | Ahmed Gasim Hospital | 10 (50.0%) |
| **Patient Status** | |  |
|  | Patient | 11 (55.0%) |
|  | Family Member | 9 (45.0%) |
| **Valvular Involvement (N=11)** | |  |
|  | Mitral valve only | 7 (63.6%) |
|  | Aortic valve and mitral valve | 2 (18.2%) |
|  | Not reported | 2 (18.2%) |
| **History of Heart Valve Surgery (N=11)** | | |
|  | Yes | 4 (36.4%) |
|  | No | 7 (63.6%) |
