# Supplementary material for: Health system and patient-level factors serving as facilitators and barriers to rheumatic heart disease care in Sudan
Source: Glob Health Res Policy. 2021 Oct 2;6:35. doi: 10.1186/s41256-021-00222-2 (PMC8486630; doi:10.1186/s41256-021-00222-2)
Supplement: Supplementary file 6 — Additional file 6. This is a supplemental table providing exemplar quotes from the qualitative portion of the study, alongside their corresponding major and minor themes. This provides a more in-depth look at the participant responses from the focus group discussions. [file 41256_2021_222_MOESM6_ESM.docx]

**Supplemental Material 6: Supplementary Tables 2A and 2B**

**Table 2A – Facilitators of RHD Treatment**

| **Domain** | **Major Theme** | **Minor Theme** | **Exemplar Quote** |
| --- | --- | --- | --- |
| **Individual** | Patient knowledge of disease process | In-depth knowledge of treatment | “If my anticoagulation levels were high I would eat some watercress [leafy vegetable] for 3 days and lower them to 2-2.5 if find they are 1-1.5 I would eat some ginger for 3 days and It will return to a 3. I find people here who are hospitalized for a month because of anticoagulation not controlled. Once they were shocked and told me my INR is 10, I said ok, just leave me for a few days in which I ate lentils and liver. After 3 days I returned, and they checked it…it was 2.5.” Participant 7, M |
|  |  | Knowledge of disease etiology | “Tonsillitis caused my disease. I take medications to prevent that [from happening again].” Participant 1, F |
|  |  | Knowledge of disease symptoms | “I used to get pricking sensations and tonsillitis, but it has now gone after the operation. Before the operation I used to get very tired.” Participant 1, F |
|  | Perception of improved symptoms with treatment | Perceived improvement of symptoms with BPG injections | “The tiredness comes before I take it, I take the injection on the 7^th^ of the month, I get the tiredness on the 3^rd^ and 4^th^. But after the injection I get better.” Participant 1, F  “When I was sick, I used to skip school for a month and continue the rest of the year…after I did this operation within 15 days I got back and I got to first grade in high school. After another 15 days I took the exams.” Participant 7, M |
|  |  | Optimism surrounding treatment options | “It is treated if one treats it, takes good nutrition and [does other things] and then [they have] hope” Participant 10, F |
|  | Positive influence of faith on attitude towards disease | No subtheme present | “Praise be to Allah, it’s all for the best, but we expect to be better after the operation.” Participant 3, F |
| **Interpersonal** | Positive influence from other’s treatment success | No subtheme present | “I see how [another patient] is living since she lives in our neighborhood. She is very active and goes out with us without being annoyed from her disease and –thanks to Allah- she has done the operation trusting Allah that she will be ok and recover. She is the one who is encouraging me to do the operation and get relieved from this stress. Praise to God she was travelling to the operation the next day and we had the wedding of her cousin in our same house, and everybody was coming to say farewell to her. She was normal and the wedding proceeded normally, and she did not suffer, God protected her.” Participant 4, M |
|  | Family support | Emotional support during treatment | “All of this was a result of my mother’s prayers. She fought really hard for me, she is my whole family to me, she spent almost 20 years at the side of my hospital bed, my other family members didn’t do anything for and they fought her.” Participant 7, M  “The antibiotics, the penicillin. When he first got the disease, he used always go to doctors and then I said to him for this disease you should do an ultrasound to show you where the disease is and you should you go to a doctor to show you the direction to go to.” Participant 5, F |
|  |  | Financial support with treatment costs | “[My family members] are all worried and keep comforting and ask us not to worry about the rent and operation money, meaning they will help us.” Participant 12, M |
|  | Motivation to undergo treatment to support family | No subtheme present | “I was really sick, and I wanted to do [treatment] to relieve my family.” Participant 7, M |
| **Systemic** | Perceived superior quality of care at referral hospital | Perceived superiority of healthcare providers at referral hospitals | “But honestly I see Ahmed Gasim hospital [a referral hospital] as one of the best hospitals and this a truth. The doctors are great and collaborative with the patients.” Participant 13, M  “Al-Shaab [referral hospital] is good as our relatives said…I am getting [the care] exactly as I had it in my mind.” Participant 9, F |
|  |  | Availability of medical treatment at referral hospitals | “[Medical treatment] is available if you can afford it. If you have money, you get treatment or else may God help you.” Participant 2, F |
|  |  | Good quality of ancillary service/cleanliness of referral hospital | “The toilets are good and the food is regular [at Al-Shaab].” Participant 10, F  “The healthcare here [Ahmed Gasim] is great, the doctors and the nurses are helpful and the bathrooms are always clean the whole 24 hours and there is no problem.” Participant 2, F |
|  | Financial assistance from institutions/groups | Financial support from the government | “The Alms chamber, the ministry of finance and the insurance gave us 450 SDG [to help pay].” Participant 5, M |
|  |  | Financial support from hospitals/pharmacies | “There are some doctors who will do [the operation] for free.” Participant 10, F  “I go to the Central Supplies [the main government drug store] and the nurses solve the problem.” Participant 8, F |
|  |  | Financial support from the community | “People help each other, there has been no time that anyone is left wanting.” Participant 10, F |

**Supplemental Table 2B – Barriers to RHD Treatment**

| **Domain** | **Major Theme** | **Minor Theme** | **Exemplar Quote** |
| --- | --- | --- | --- |
| **Individual** | Injection pain | No subtheme present | “Yes, it was painful and that was also a reason of stopping it, but I didn’t realize [stopping it] would deteriorate me to this stage.” Participant 7, M |
|  | Misconception of disease/poor understanding of disease | Misunderstanding of cause of disease | “I mean drinking too much coffee and cigarettes. All these things cause complications and affect the heart because the heart is the main building which pushes the body. Also, too much stress affects the heartbeat, which affects the heart arteries.” Participant 9, F  “Participant: They asked me if I ever got tonsillitis, I told them no. But I had high blood pressure since my twenties, and I am on the medication till this July when I got worse.  Facilitator: So, do you think that high blood pressure is the cause of the disease?  Participant: Yes, he diagnosed me with this valve disease and told me that we should look for the cause of this high blood pressure since I got it at a young age.” Participant 3, F |
|  |  | Poor understanding of disease management | “I try to live with it by avoiding things that harm me. Avoiding harmful things like cigarettes, coffee. All these things I try to avoid as much as I can.” Participant 9, F |
| **Interpersonal** | Poor communication between provider and patient | Poor communication regarding diagnosis | “I discovered I had this disease 8 years ago. The doctor didn’t tell me there was something wrong with my valve and I found out by accident when I looked into my file. I then told my mother who said we should go to the doctor, and we went to him and she asked him “Does my son have a valve [disease]?” He asked her, “Who told you?” and she just repeated her question adding if he has valve [disease] we want to refer him to Khartoum.” Participant 7, M |
|  |  | Poor communication regarding treatment | “I had two [diseased] valves and at the hospital they would just widen the one valve. They would just widen and widen. I had two [affected] valves and I wanted to know if it’s okay to widen one valve and leave the other. Why hide [instead of just] telling me that you can’t do both? But they insisted on the [widening) operation. The first time they said they widened a valve, I wondered are they going to widen both valves? I left and came a year later, they said they would do a widening operation but after 9-10 days I got even worse, so I just paid for nothing. After this operation I laid in bed for 4 years and couldn’t move. I’m from Al-Duwaim and in the operation there was hemorrhage that they sealed shut and they didn’t tell my kin about it. Eventually they made us pay for the operation. There were so many shortages and mistakes in the past that I could have avoided.” Participant 7, M  “Also getting a definite date for surgeries is important. Especially for people who live in far areas. Meeting a doctor several times and not getting a definite date is frustrating. It would be very much comforting to be assigned a date at an early time.” Participant 2, F |
|  | Poor relationship between patients and providers | Patient Perspective | “First the patient should be cool and not speak rudely to the doctors. This is an important point because some patients and their companions are tense and fight with the nurses and doctors. The second thing is you should follow orders and help the nurses and doctors in the treatment and follow their advice if they say take the medicine at 6. By 6, you should take it.” Participant 9, F |
|  |  | Provider Perspective | “This is the problem with Sudan. Even if your father is an illiterate farmer and he tells you to do something you would do it. Even if you know it’s wrong and you won’t ask because it would be considered rude, the same applies to doctors. When an operation can’t be done in Sudan but can be done abroad, the doctors assume that 99% of the public can’t afford to do it outside the country. They would decide to do something else and they would make the decision alone without telling you.” AE, Researcher |
|  | Negative impact/burden on family members | No subtheme present | “But you know the person who is sick is the problem for the whole house.” Participant 17, F  “Patient’s Mother: Honestly we’re patient [regarding interruptions of observing Ramadan]. Last thing before we got her here at Ramadan, she used to stay up all night in agony and sometimes vomiting, I ask repeatedly and urged her to go to the Emergency Room, but she says there is nothing I’m fine [because of Ramadan]. I keep telling [the patient] ‘you must go so you can have painkillers there’. Thank God, they helped her and now she is hospitalized here.” Participant 6, F |
| **Systemic** | Poor quality of patient education by providers | No subtheme present | “Do you know I worked in this hospital? After three months I asked them ‘why didn’t you tell me?’ They told me that, ‘I am semiliterate and won’t understand and lack knowledge and can’t deal with the warfarin and the anticoagulation and after a year we will do you the mechanical valve for you.’ I didn’t pay again if I knew I had two valves to be changed [then] I would’ve told them that I want to work there and after 10 years they would’ve done the operation for free.” Participant 7, M  “The treatment is good and the services are good but there is one thing: There is a trainer who comes and shows you, but until now no doctor showed me.” Participant 10, F |
|  | Significant costs associated with treatment | Significant costs of medical treatment/operation | “You tell me you have an operation after 10 years for 50 million. Where can I pay it from? So I told them to give me a job as they knew this and so I worked at the cafeteria and after 3 months I took control of the cafeteria and worked for 7-8 years. Then they said I should pay to have the operation [even after] working in the hospital for 10 years.” Participant 7, M  “But when you go to the doctor and it is not his specialty he should send you to someone whose specialty it is and not just write prescriptions and make you lose money. He does not know your [financial] situation and you go to the pharmacy and they tell you to pay 300 [SDG], pay 400, pay 200 and he [the doctor] takes 200 or 300 and it isn’t even his specialty.” Participant 18, M  “Money is fate. God divides it, there are poor and there are wealthy so I don’t choose. If I am afraid of something, I don’t lose anything if I have nothing.” Participant 20, M |
|  |  | Significant costs of non-medical amenities (food, lodging, transportation) | “It’s just the harsh questioning from the doormen and not allowing homemade meals in the hospitals and they are not providing meals for the public sector. They gave us breakfast when we were in the private sector.” Participant 4, M  “The apartment rent [near the referral hospital] cost 6 thousand SDG [per month].” Participant 6, F |
|  | Lack of transportation to appropriate healthcare facility | No subtheme present | “Transportation is a major problem between the hospitals and from the house to the hospital. Autumn is 3-4 months and because of the rain you can’t leave your house and get medical care, there is also the issue of the bus fee.” Participant 7, M |
|  | Lack of trust in local health systems | Lack of adequate healthcare (personnel/surgical equipment) at local hospitals/facilities | “It would improve if treatment is available nearby. If not a hospital, a prepared health center. Even if you are diagnosed somewhere else, you can go there for follow-up and to take your medicines.” Participant 7, M  “One of the oldest places in Sudan with no laboratories, no specialists, no registrars. There are good doctors and each one stays a year or so in the hospital, so [there is no continuity of care].” Participant 9, F  “Now the operation should have been in June but because of the money [it was postponed]. Now the hospital is closed [for repairs] and she can’t do the operation.” Participant 18, M |
|  |  | Improper use of referrals preventing adequate care | “What I [can’t understand] is that one of the consultants I saw was a chest specialist and he should have told me to see a heart doctor to see what is happening to your heart or to do an echocardiography or something like that.” Participant 9, F  “With all due respect, the doctors should advise people who come to them. They should examine them and know the disease is not theirs [specialty wise]. But nobody advised him, and they just write him [a prescription] to go the pharmacy. With all due respect the doctors are like car mechanics: one thing is wrong in your car they tell you it is another.” Participant 13, M |
|  |  | Misdiagnosis at local facility | “4 months I spent rotating between consultants. With all due respect to you doctors, these are the papers in my file, all the 5 doctors I saw none of them told me to see a heart specialist or told me to do tests. It was only by the grace of God that I was in a pharmacy buying medicines when I met a doctor who told me to do exercise. I was doing the first exercise session when I felt stabbing pain, the exercise was with machines and he [the sports trainer] told me [patient], ‘you have to see a heart doctor’. So the specialists told me I had nothing and labeled me as tuberculosis and all these pills caused me complications and increased my plight.” Participant 16, F  “Her illness became when we went to the doctor, and he didn’t give her good medicine. The medicine itself causes diseases.” Participant 18, M |
|  |  | Poor quality of ancillary services (nursing) in local hospitals | “In nursing there are some clever people, and they are in the right place but there are those who are in the wrong place and these maltreat people. The responsible people in the ministry of health when they appoint people should send them for training for two to three years not two or three months and come back to the hospital and they still have lessons and other things [training] left.” Participant 15, F |
|  | Long wait-times for treatment | Delay in diagnosis | “There is a lot of pressure on the emergency department, they see more than a 100 [patients] and may put patients in beds and the floor. I met a fair colored female doctor when I came from the morning, I was sitting on a chair, and we lay down on the stairs till the sunset [before being seen].” Participant 19, F |
|  |  | Delay to see specialist | “At the hospital… you have to wait. You get to the ER where they sedate you and after that you meet the specialist on Monday or someday and so you find that you waste time.” Participant 7, M |
|  |  | Delay to get surgery | “The operation takes a lot of time [before occurring]. For one patient, it was one month, but for [me] it was one year.” Participant 3, F |
|  | Perception of poor quality care by younger/less experienced providers | No subtheme present | “With all due respect, doctors are different, some of them are useless and don’t know anything. They are carrying stethoscopes and examine you and write a prescription which do not make you better and even make you worse. In these hospitals there should be doctors with good certificates, who are well trained and experienced not those who just graduated 2 or 3 months and entered a hospital. These won’t treat you, they will make you more sick.” Participant 14, M |
|  | Perception of higher quality care in other settings | No subtheme present | “Now I have a [insurance] card and I go for treatment in Asia at a private hospital. They are good and cure me.” Participant 16, M  “All these people are sick, 95% of Sudanese are sick but don’t know where to go and get treated. All people now go to Egypt, they take a bus for 600 SDG going, 600 SDG going back and take 10 or 20 thousand SDG and go and get treated well and return back.” Participant 15, F |
